# Dietary Valine/Isoleucine Ratio Impact Carcass Characteristics, Meat Edible Quality and Nutritional Values in Finishing Crossbred Duroc × Landrace × Yorkshire Pigs With Different Slaughter Weights

**DOI:** 10.3389/fnut.2022.899871

**Published:** 2022-07-11

**Authors:** Doudou Xu, Yubo Wang, Xin Zhang, Enfa Yan, Linjuan He, Lu Wang, Chenghong Ma, Pengguang Zhang, Jingdong Yin

**Affiliations:** State Key Laboratory of Animal Nutrition, College of Animal Science and Technology, China Agricultural University, Beijing, China

**Keywords:** dietary ratio of valine to isoleucine, slaughter weight, water holding capacity, fatty acid composition, amino acid profile

## Abstract

The aim of this study was to investigate effects of dietary ratio of valine to isoleucine [R(V/I)] on carcass characteristics and meat quality of finishing pigs and whether slaughter weight influence the effect. We carried out a 2 × 3 factorial trial with two slaughter weight (105 vs. 130 kg) and three R(V/I) (0.58, 1.23, and 2.60 at 75–100 kg body weight, and 0.70, 1.24, and 2.39 at 100–135 kg body weight for L-, N- and H-R (V/I), respectively). Data show that increasing slaughter weight significantly increased meat color (a*_45 min_ and b*_45 min_), drip loss and shear force (*P* < 0.05). Meanwhile, increasing slaughter weight reduced sarcomere length, the proportion of protein-bound water, and most kinds of muscular total amino acid contents except for tryptophan and arginine, while increased contents of muscular free lysine, tryptophan, leucine, isoleucine, valine, alanine, and arginine in the M. longissimus thoracis (*P* < 0.05). Health lipid indices based on fatty acid composition of intramuscular lipid were improved as the slaughter weight increased (*P* < 0.05). Notably, pigs received N-R (V/I) diet improved carcass traits in terms of thinner backfat thickness and higher fat-free lean index, as well as increased meat flavor-contributing amino acids at the cost of reduced intramuscular fat content and increased shear force of cooked meat compared with the pigs fed L-R (V/I) and H-R(V/I) diets (*P* < 0.05). H-R (V/I) diet decreased ultimate pH value and sarcomere length of the skeletal muscle but increased the proportion of free water (*T*_23_), consequently, increased drip loss and cooking loss of fresh meat in pigs (*P* < 0.05). In conclusion, both slaughter weight and dietary ratio of valine to isoleucine exerted significant impacts on carcass characteristics, meat quality and nutrition values. In particular, carcass traits and meat color of lighter pigs were more susceptible to the influence of dietary R (V/I) relative to heavier pigs.

## Introduction

Branched-chain amino acids (BCAAs), including leucine, isoleucine and valine, play a vital role in skeletal muscle protein synthesis and metabolism. The amount of dietary leucine usually exceeds the requirement of pigs in commercial diets, while isoleucine and valine are regarded as limiting amino acids following lysine, methionine, threonine and tryptophan for obtaining optimal growth performance of pigs. The considerable wide range of BCAA combinations (1:0.25:0.25∼1:0.75:0.75, leucine:isoleucine:valine) has been reported to increase lipid accumulation in skeletal muscle and promote protein metabolism and muscle growth in growing pigs (1, 2). In our previous study, we found that dietary supplementation of extra isoleucine can enhance intramuscular fat accumulation and monounsaturated fatty acids (MUFA) synthesis in skeletal muscle through depressing the phosphorylation of AMPKα-ACC and stimulating the expression and activity of SCD in finishing pigs (3). Furthermore, we found the coordination existed between dietary valine and isoleucine on backfat thickness and water holding capacity of fresh meat in finishing pigs (4). Therefore, we supposed that the dietary ratio of valine to isoleucine [R(V/I)] might impose a significant impact on carcass characteristics and meat quality in finishing pigs.

The compositions of muscular free amino acids and fatty acids are of great interest in meat industry since they are important for meat edible quality (5, 6). We speculate that BCAA especially isoleucine and valine might be involved in the regulation of amino acid and fatty acid profiles of fresh meat as they promote protein synthesis and intramuscular fat (IMF) formation (3, 7).

Besides, slaughter weight is an important factor determining the economic profitability of pork production. Presently, pig slaughter weight especially in China trends to increase from 110 kg to more than 130 kg in order to reduce production costs and earn more margin profit (8). However, some studies have shown disadvantages caused by increased slaughter weight, such as reduced feed conversion efficiency, excessive backfat thickness and decreased lean rate of pigs (9, 10).

The effect of dietary nutrient level on carcass characteristics and meat quality may be influenced by slaughter weight (11). However, there are few studies concerning effects of dietary BCAA on carcass characteristics and meat quality and nutrition values between different slaughter weights of finishing pigs. Therefore, the objective of this experiment was to test the interactive effects, if any, of dietary R(V/I) and slaughter weight on the fatty acid profile and free amino acid composition of fresh meat in finishing pigs.

Together, given economic benefit in the swine industry, the nutrition and eating quality of meat, we aimed to explore the effects of dietary ratio of valine to isoleucine [R(V/I)] on carcass characteristics and meat quality of finishing pigs and whether slaughter weight influence the effect.

## Materials and Methods

### Ethics Statement

All procedures conducted in the present study were approved by the Institutional Animal Care and Use Committee of China Agriculture University (ID: SKLAB-B-2010-003).

### Experiment Design, Animals and Experimental Diets

Fifty-four crossbred castrated male pigs (Duroc × Landrace × Yorkshire) with an initial body weight of 74.1 ± 1.3 kg were allocated into three dietary treatments in a randomized complete block design based on the initial body weight (BW), with six replicates (pens) per treatment and three piglets per pen. All the pigs were housed in pens (1.8 × 2.1 m^2^) with 50% of slatted floors in an environmentally controlled room in FengNing Swine Research Unit of China Agricultural University (Academician Workstation in Chengdejiuyun Agricultural and Livestock Co., Ltd., Fengning County, Hebei Province, China).

The experimental diets were formulated to be isoenergetic and to meet the nutritional requirements for pigs of 75–100 kg and 100–135 kg BW (12), respectively. Experimental diets were formulated to be equivalent in the amounts of standard ileal digestible (SID) essential AA except for isoleucine and valine. The levels of isoleucine and valine were formulated according to experiment design. The composition and nutrient levels of the experimental diets are shown in Table 1. At the beginning, pigs were fed with diets of 75–100 kg BW. The three experimental diets were formulated with R (V/I) as follows: L-R (V/I) = 0.58, (SID valine = 0.31%, SID isoleucine = 0.53%); N-R (V/I) = 1.23, (SID valine = 0.48%, SID isoleucine = 0.39%); H-R (V/I) = 2.60, (SID valine = 0.65%, SID isoleucine = 0.25%). While the average BW of pigs exceeded 100 kg (actual 104.41 kg), pigs close the average BW of pen were selected to be humanly slaughtered (*n* = 6). The remaining 36 pigs shift to diets for 100–135 kg BW (Table 1) until BW approached 130 kg (actual 131.52 kg). The R (V/I) of the three dietary treatments were formulated as follows: L-R (V/I) = 0.70 (SID valine = 0.31%, SID isoleucine = 0.44%), N-R (V/I) = 1.24 (SID valine = 0.41%, SID isoleucine = 0.33%), H-R (V/I) = 2.39 (SID valine = 0.55%, SID isoleucine = 0.23%). During feeding trials, pigs had *ad libitum* access to the diets and clean drinking-water for 63 days. At the end of experiment, one pig from each pen was humanly slaughtered. The pigs slaughtered at two stages were subjected to assessment of carcass characteristics and meat quality as well as meat nutritional composition.

**TABLE 1:** The composition and nutrient content of the experimental diets provided for finishing pigs (%)*^a^*.

Item*^a^*	75–100 kg BW			100–135 kg BW		
	L-R (V/I)	N-R (V/I)	H-R (V/I)	L-R (V/I)	N-R (V/I)	H-R (V/I)
**Ingredients**						
Corn	88.70	88.70	88.70	90.74	90.75	90.75
Soybean meal	2.90	2.90	2.90	1.50	1.50	1.50
Wheat bran	2.50	2.50	2.50	2.26	2.26	2.26
Soybean oil	1.80	1.80	1.80	1.80	1.80	1.80
Limestone	0.96	0.96	0.96	0.96	0.96	0.96
Dicalcium phosphate	0.40	0.40	0.40	0.40	0.40	0.40
Salt	0.35	0.35	0.35	0.35	0.35	0.35
L-Lysine HCl	0.63	0.63	0.63	0.52	0.52	0.52
DL-Methionine	0.12	0.12	0.12	0.07	0.07	0.07
L-Threonine	0.23	0.23	0.23	0.19	0.19	0.19
L-Tryptophan	0.08	0.08	0.08	0.07	0.07	0.07
L-Isoleucine*^b^*	0.26	0.14	0.01	0.21	0.10	0.00
L-Valine*^b^*	0.00	0.15	0.30	0.00	0.10	0.23
L-Histidine⋅HCl*^b^*	0.10	0.10	0.10	0.06	0.06	0.06
L-Phenylalanine*^b^*	0.09	0.09	0.09	0.04	0.04	0.04
L-Alanine*^b^*	0.21	0.18	0.16	0.16	0.16	0.13
50% Choline chloride	0.08	0.08	0.08	0.08	0.08	0.08
Sweetener	0.08	0.08	0.08	0.08	0.08	0.08
Phytase	0.01	0.01	0.01	0.01	0.01	0.01
Premix*^c^*	0.50	0.50	0.50	0.50	0.50	0.50
**Analyzed nutrient levels**						
Crude protein	10.92	10.39	10.98	9.84	9.97	10.11
Lysine	0.74	0.79	0.78	0.68	0.70	0.61
Methionine + cysteine	0.44	0.45	0.45	0.38	0.39	0.39
Threonine	0.48	0.52	0.49	0.40	0.46	0.42
Tryptophan	0.17	0.17	0.17	0.14	0.15	0.15
Isoleucine	0.53	0.43	0.30	0.43	0.38	0.26
Leucine	0.95	1.00	0.93	0.82	0.97	0.86
Valine	0.39	0.59	0.66	0.41	0.50	0.63
**Calculated nutrient levels**						
DE, MJ/kg	14.19	14.19	14.19	14.24	14.24	14.24
ME, MJ/kg	13.92	13.92	13.92	13.81	13.81	13.81
Crude protein	10.83	10.83	10.83	10.01	10.01	10.01
**Standardized ileal digestible amino acids**						
Lysine	0.73	0.73	0.73	0.61	0.61	0.61
Methionine + cysteine	0.42	0.42	0.42	0.36	0.36	0.36
Threonine	0.46	0.46	0.46	0.40	0.40	0.40
Tryptophan	0.13	0.13	0.13	0.11	0.11	0.11
Isoleucine	0.53	0.39	0.25	0.44	0.33	0.23
Leucine	0.89	0.89	0.89	0.87	0.87	0.87
Valine	0.31	0.48	0.65	0.31	0.41	0.55
R (V/I)	0.58	1.23	2.60	0.70	1.24	2.39

*^a^L-R (V/I), low ratio of valine to isoleucine; N- R (V/I), normal ratio of valine to isoleucine; H-R (V/I), high ratio of valine to isoleucine. ^b^Purchased from Beijing Jiakangyuan Technology Development Company, Beijing, China. ^c^The premix provided the following per kg of diets: vitamin A, 6,000 IU; vitamin D_3_, 2,400 IU; vitamin E, 20 IU; vitamin K_3_, 2 mg; vitamin B_1_, 0.96 mg; vitamin B_2_, 4 mg; vitamin B_6_, 2 mg; vitamin B_12_, 0.012 mg; biotin, 0.04 mg; folic acid 0.40 mg; pantothenic acid 11.2 mg; nicotinic acid 22 mg; Cu, 120 mg; Fe, 76 mg; Mn, 12 mg; Zn, 76 mg; I, 0.24 mg; Se, 0.40 m.*

### Sample Collection

Before the slaughter, blood samples were collected from the precaval vein of pigs close to the average BW of each pen (*n* = 6) after overnight starvation for 16 h. Serum was separated and stored at −20°C for further analysis. Pigs were transported to local abattoir. After rest at least 4 h, the pigs were electrically stunned, exsanguinated and eviscerated according to the standard commercial procedure. Moreover, at 30 min postmortem, chops of the M. longissimus thoracis from the 10th to 12th ribs of each left carcass were separated and trimmed off extra-muscular fat and connective tissues. After the determination of meat color and pH at 45 min (pH_45 min_), these chops were vacuum-packed at 4°C to measure subsequently meat quality, including muscle pH at 24 h (pH_24 h_), drip loss, cooking loss and shear force as well as NMR transverse relaxation (T2) measurement. About 100 g of chops were stored at –20°C for measurement of contents of moisture, crude protein and IMF, as well as amino acid and fatty acid composition. For myofibril ultrastructure examination, the M. longissimus thoracis between the 9th and 10th rib was cut to 1 mm^3^ and stored immediately in fixing solution (2.5% glutaraldehyde phosphate buffer saline, pH 7.2).

### Carcass and Meat Quality Evaluation

Dressing percentage was calculated by dividing the hot carcass weight by slaughter weight. Backfat depth at the thickest shoulder, last rib, 6th to 7th rib, 10th rib, and the last lumbar vertebra were recorded. Fat-free lean index = 50.767 + [0.035 × hot carcass weight (Ib)] – [8.979 × the last rib fat thickness (in.)] (NPPC, 1994) (13). Loin eye area (cm^2^) = loin eye height (cm) × width (cm) × 0.7).

At 45 min postmortem, meat color (L*, a*, b*) was measured with a Minolta Chroma Meter (Minolta Chroma Meter Measuring Head CR-410 Minolta, Osaka, Japan), pH_45 min_ value was measured with a SPK pH meter (pH-star, DK2730, Herlev, Denmark). Meanwhile, the pH_24 h_ value was determined at 24 h postmortem in 4°C chilling room.

Drip loss was determined by a suspension method and calculated as follows: drip loss (%) = [(initial weight-final weight)/initial weight] × 100. The pork cooking loss and shear force was measured. Briefly, the M. longissimus thoracis chops were cooked to an internal temperature of 70°C for 30 min in 75°C thermostatic water bath. Chops were allowed to cool to room temperature, wiped with absorbent paper to remove residual moisture and reweighed to calculate cooking loss. Then the cooked chops were cut to 1 × 1 × 3 cm^2^ parallel to the muscle fiber orientation to measure the tenderness using a digital-display muscle tenderness meter (C- LM3B, Tenova, Harbin, China). Twelve replicates of each sample were measured.

Samples of the M. longissimus thoracis were cut into 2–3 mm thin slices and freeze-dried using vacuum frozen dryer (Freezone 4.5™, Labconco Corp., Kansas City, MO, United States) and ground into powder for analysis of crude protein, IMF, fatty acid and amino acid. The IMF content was analyzed by Soxhlet petroleum-ether extraction. The crude protein content was measured according to Association of Analytical Chemists methods (14).

### Nuclear Magnetic Resonance Transverse Relaxation (T2) Measurement

Low field NMR transverse relaxation was measured as described previously (4). Briefly, approximately 3 g of meat from the center of each sample were analyzed using a Niumag Pulsed NMR analyzer (PQ001, Niumag Corporation, Shanghai, China) operating at a resonance frequency for protons of 23 MHz at 32°C (15, 16). Three forms of water distribution in fresh meat including protein-bound water (a minor component between 1 and 10 ms, *T*_21_), immobilized water (a major component between 30 and 100 ms, *T*_22_) and free water (a much weaker component between 100 and 500 ms, *T*_23_).

### Myofibril Ultrastructure

As previously described (17), 2.5% glutaraldehyde-fixed isolated myofibrils were fixed with 1% osmium tetroxide (OsO4) for 2 h, and then dehydrated with ethanol gradient, followed by embedded with resin. After set in a 60°C incubator for 48 h, the solidified tissue was made into an ultrathin section, and then subjected to double electron staining of uranyl acetate-lead citrate. After washing with water, myofibril ultrastructure was observed under a transmission electron microscope (H-7500, HITACH Corporation, Japan), and sarcomere length, the distances between Z lines, representing a measure of muscle contraction was measured.

### The Compositions of Amino Acids and Fatty Acids in Fresh Meat

The free amino acid concentration in the M. longissimus thoracis was analyzed using ultra high-performance liquid chromatograph (Waters ACQUITY UPLC I-Class, Waters, United States) and high-resolution mass spectrometer (Q-Exactive, Thermo Fisher, United States) as previously described (18). About 300 mg muscle sample were dissolved in water with methanol (water: methanol = 2:8, v/v). After ultrasonic treatment for 5 min, the sample was placed at room temperature for 1 min, and the above operation was repeated for six times. The sample was then placed on ice for 2 h and centrifuged at 10,000 *g*, 4°C for 10 min to collect the supernatant.

Total amino acid (mainly protein-bound amino acids) profile of the M. longissimus thoracis was determined based on the standard methods in AOAC (14).

The composition of fatty acids of the M. longissimus thoracis was analyzed as described previously (19). About 150 mg lyophilized muscle sample was added with 4 ml chloracetyl methanol (1:10, v/v), 1 mL n-hexane and 1 mL internal standard FA solution (1 mg/mL C11:0). The mixture was then kept in a water bath at 75°C for 2 h. After cooling to room temperature, the mixture was added with 5 mL carbonate solution (70 g/L) and centrifuged at 800 *g* for 3 min. The supernatant was analyzed by the Gas Chromatography (HP 6890 series, Hewlett Packard, Avondale, PA, United States).

### Lipid Health Quality Indexes

The health quality of the lipid components of muscle was assessed by calculating: (1) the index of atherogenicity (IA), (2) the index of thrombogenicity (IT), and (3) the ratio between the hypocholesterolemic and hypercholesterolemic fatty acids (HH).

The index of atherogenicity (IA) indicates the relationship between the sum of the main saturated fatty acids and that of the main classes of unsaturated ones. Saturated fatty acids are considered pro-atherogenic (favoring the adhesion of lipids to cells of the immune and circulatory system). However, unsaturated fatty acids are qualified as anti-atherogenic (inhibiting the aggregation of plaque and diminishing the levels of esterified fatty acid, cholesterol, and phospholipids, thereby preventing the appearance of micro- and macro-coronary diseases).

The index of thrombogenicity (IT), defined as the ratio of the pro-thrombogenetic (saturated) to the anti-thrombogenetic (unsaturated) fatty acids, shows the tendency to form clots in the blood vessels.

The following equations were used to calculate these indexes:

- Atherogenic Index (20)


$$I\,A=\frac{\left(4\times C\,14:0+C\,16:0+C\,18:0\right)}{\left(\Sigma \,M\,U\,F\,A+\Sigma \,n\,6\,P\,U\,F\,A+\Sigma \,n\,3\,P\,U\,F\,A\right)}$$

- Thrombogenic Index (20)


I⁢T

$$=\frac{\left(\mathrm{C14}:0+C16:0+\mathrm{C18}:0\right)}{\begin{array}{c} (0.5\times \Sigma \mathrm{MUFA}+0.5\times \Sigma n6\mathrm{PUFA}+3\times \Sigma n3\mathrm{PUFA}\\ +\Sigma n3\mathrm{PUFA}/\Sigma n6PUFA) \end{array}}$$

- Ratio between hypocholesterolemic and hypercholesterolemic fatty acids (21)


$$\mathrm{HH}=\frac{\begin{array}{c} (\mathrm{C18}:1n9+\mathrm{C18}:2n6+\mathrm{C20}:4n6+\mathrm{C18}:3n3+\mathrm{C20}:5n3\\ +\mathrm{C22}:5n3+\mathrm{C22}:6n6) \end{array}}{\left(\mathrm{C14}:0+\mathrm{C16}:0\right)}$$

where Σ = Summatory, MUFA = monounsaturated fatty acids, and PUFA = polyunsaturated fatty acids.

### Statistical Analyses

Data were analyzed using the mixed procedure SAS 9.2 (SAS Institute Inc., Cary, NC, United States) following a completely randomized design according to a 3 (R(V/I)) × 2 (slaughter weights) factorial arrangement with individual pig as the experimental unit. The statistical model included the fixed effects of the R(V/I), slaughter weight and their interaction and the pig as random effect of the experiment. When *P*-value for interaction terms was above 0.05, the interaction was excluded from the statistical model. If the interaction was significant, Tukey’s test was used to compare treatment means within a slaughter weight. Differences were considered significant when *P* < 0.05.

## Results

### Carcass Traits

As shown in Table 2, carcass weight and loin eye area of pigs were not altered by the dietary R (V/I). Pigs slaughtered at 130 kg BW had thicker backfat thickness compared with pigs slaughtered at 105 kg BW (*P* < 0.05). Pigs offered N-R (V/I) diet had the thinnest backfat thickness at the shoulder, last rib, and highest fat-free lean index among all pigs slaughtered at 105 kg BW (*P* < 0.05). In contrast, no effect of R (V/I) on the last rib fat thickness and fat-free lean index was observed in pigs slaughtered at 130 kg BW. Significant interactions existed between R (V/I) and slaughter weight on the last rib fat thickness and fat-free lean index.

**TABLE 2:** Effect of dietary R (V/I) and slaughter weight on the carcass traits in finishing pigs (*n* = 6).

Items	105 kg			130 kg			*P*-value		
	L-R (V/I)	N-R (V/I)	H-R (V/I)	L-R (V/I)	N-R (V/I)	H-R (V/I)	SW	R (V/I)	SW × R (V/I)
Carcass weight, kg	80.27 ± 4.25	78.12 ± 2.05	82.13 ± 1.04	100.82 ± 0.62	100.77 ± 2.37	99.10 ± 1.50	<0.01	0.40	0.02
Dressing percentage, %	75.80 ± 0.65	76.71 ± 1.23	77.16 ± 0.63	76.81 ± 0.68	76.99 ± 0.65	76.79 ± 0.50	0.39	0.26	0.28
**Subcutaneous backfat depth, cm**									
Shoulder fat thickness	3.69 ± 0.27	3.39 ± 0.07	3.62 ± 0.35	4.45 ± 0.10	3.88 ± 0.20	4.44 ± 0.28	<0.01	<0.01	0.25
The last rib fat thickness	2.06 ± 0.14	1.76 ± 0.05	2.49 ± 0.08	2.33 ± 0.12	2.45 ± 0.24	2.53 ± 0.22	<0.01	<0.01	<0.01
The last lumber vertebra	1.45 ± 0.23	1.31 ± 0.01	1.38 ± 0.30	1.93 ± 0.13	1.97 ± 0.24	1.78 ± 0.35	<0.01	0.57	0.41
The 6th to 7th rib fat thickness	2.32 ± 0.18	2.20 ± 0.19	2.32 ± 0.24	3.11 ± 0.23	2.70 ± 0.19	3.16 ± 0.32	<0.01	0.03	0.28
The 10th rib fat thickness	1.74 ± 0.18	1.84 ± 0.01	2.02 ± 0.26	2.02 ± 0.11	2.06 ± 0.27	2.05 ± 0.43	0.04	0.35	0.45
Loin eye area, cm^2^	39.78 ± 2.16	40.69 ± 1.25	42.17 ± 1.67	44.10 ± 2.02	40.95 ± 4.25	38.97 ± 4.25	0.63	0.47	0.01
Fat-free lean index, %	49.71 ± 0.16	50.45 ± 0.18	48.42 ± 0.34	50.58 ± 0.37	50.46 ± 0.37	50.17 ± 1.55	<0.01	<0.01	0.02

*L-R (V/I), low ratio of valine to isoleucine; N- R (V/I), normal ratio of valine to isoleucine; H-R (V/I), high ratio of valine to isoleucine; SW means slaughter weight.*

### Meat Quality

As shown in Table 3, with the slaughter weight increased, meat quality in terms of a*_45 *min*_ (*P* = 0.03), b*_45 min_, pH_24 *h*_ value, shear force, marbling score and drip loss as well as intramuscular fat content significantly increased (*P* < 0.01), while crude protein level decreased (*P* < 0.01). Dietary R (V/I) significantly affected most indices of meat quality, especially in N-R (V/L) treatment, shear force (*P* = 0.02) was the highest while intramuscular fat content (*P* = 0.05) was the lowest among dietary treatments. Additionally, significant interaction between slaughter weight and dietary R (V/I) was observed on pH values (*P* < 0.01), a*_45 *min*_ (*P* = 0.03) and marbling score (*P* = 0.02).

**TABLE 3:** Effect of dietary R (V/I) and slaughter weight on the meat quality and proximate analysis of *longissimus dorsi* in finishing pigs (*n* = 6).

Items	105 kg			130 kg			*P*-value		
	L-R (V/I)	N-R (V/I)	H-R (V/I)	L-R (V/I)	N-R (V/I)	H-R (V/I)	SW	R (V/I)	SW × R (V/I)
pH_45 *min*_	6.27 ± 0.13	6.34 ± 0.12	6.18 ± 0.08	6.30 ± 0.10	6.23 ± 0.09	6.38 ± 0.09	0.21	0.98	<0.01
pH_24 *h*_	5.58 ± 0.02	5.63 ± 0.04	5.46 ± 0.04	5.57 ± 0.01	5.59 ± 0.01	5.59 ± 0.05	0.03	<0.01	<0.01
L*_45 *min*_	47.66 ± 0.92	46.02 ± 0.97	46.32 ± 1.36	47.48 ± 1.59	47.13 ± 2.11	46.39 ± 2.18	0.53	0.16	0.58
a*_45 *min*_	15.66 ± 0.14	16.90 ± 0.42	16.60 ± 0.56	18.38 ± 1.09	18.27 ± 0.52	18.63 ± 0.42	<0.01	0.04	0.03
b*_45 *min*_	2.39 ± 0.32	2.12 ± 0.25	1.99 ± 0.27	3.15 ± 0.31	2.78 ± 0.50	2.89 ± 0.37	<0.01	0.04	0.69
Drip loss, %	1.82 ± 0.21	2.01 ± 0.27	3.55 ± 0.69	2.59 ± 0.44	2.33 ± 0.13	3.87 ± 0.40	<0.01	<0.01	0.30
Cooking loss, %	25.86 ± 1.38	23.64 ± 1.37	27.46 ± 1.43	25.57 ± 0.63	25.21 ± 0.90	26.97 ± 0.73	0.49	<0.01	0.06
Shear force, N	40.77 ± 1.83	41.22 ± 2.24	38.89 ± 2.90	42.65 ± 1.13	47.25 ± 3.23	42.84 ± 4.62	<0.01	0.02	0.23
Marbling score	1.20 ± 0.19	1.25 ± 0.22	1.42 ± 0.38	1.83 ± 0.20	1.25 ± 0.27	1.50 ± 0.32	0.01	0.06	0.02
Crude protein, %	24.06 ± 0.68	23.76 ± 0.53	23.64 ± 0.49	21.77 ± 2.16	22.08 ± 0.64	22.67 ± 0.66	<0.01	0.81	0.32
Intramuscular fat, %	2.85 ± 0.26	2.27 ± 0.47	3.07 ± 0.45	3.69 ± 0.60	3.19 ± 0.52	3.62 ± 0.89	<0.01	0.05	0.76

*L-R (V/I), low ratio of valine to isoleucine; N- R (V/I), normal ratio of valine to isoleucine; H-R (V/I), high ratio of valine to isoleucine; SW means slaughter weight.*

### Nuclear Magnetic Resonance Transverse Relaxation (T2)

**FIGURE 1:**
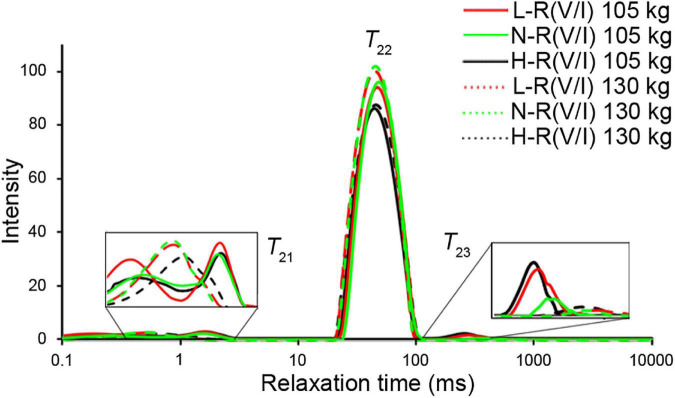
Relaxometry time of LF-NMR (*n* = 6).

As shown in Table 4 and Figure 1, the proportions of bound water (*T*_21_) and free water (*T*_23_) decreased with the slaughter weight increased, while the proportion of immobilized water (*T*_22_) increased (*P* < 0.01). Pigs fed with L-R (V/I) diets had the highest proportion of bound water and pigs fed with H-R (V/I) diets had the highest proportion of free water, while pigs fed N-R (V/L) had the lowest proportion of free water and highest proportion of immobilized water (*P* < 0.01). The significant interaction between slaughter weight and R (V/I) was found on the proportions of bound water (*P* = 0.02) and immobilized water distribution (*P* = 0.03).

**TABLE 4:** Effect of dietary R (V/I) and slaughter weight on the *T*_2_ peak area ratio of *longissimus dorsi* in finishing pigs (*n* = 6).

Items	105 kg			130 kg			*P*-value		
	L-R (V/I)	N-R (V/I)	H-R (V/I)	L-R (V/I)	N-R (V/I)	H-R (V/I)	SW	R (V/I)	SW × R (V/I)
*T*_21_	5.75 ± 0.33	4.95 ± 0.41	5.01 ± 0.50	3.96 ± 0.18	3.91 ± 0.03	3.75 ± 0.11	<0.01	<0.01	0.02
*T*_22_	93.26 ± 0.43	94.30 ± 0.77	93.58 ± 0.63	95.96 ± 0.11	96.00 ± 0.17	95.93 ± 0.15	<0.01	0.02	0.03
*T*_23_	1.00 ± 0.46	0.48 ± 0.20	1.21 ± 0.62	0.16 ± 0.02	0.13 ± 0.10	0.29 ± 0.08	<0.01	<0.01	0.09

*L-R (V/I), low ratio of valine to isoleucine; N- R (V/I), normal ratio of valine to isoleucine; H-R (V/I), high ratio of valine to isoleucine; SW means slaughter weight.*

### Myofibril Ultrastructure

**FIGURE 2:**
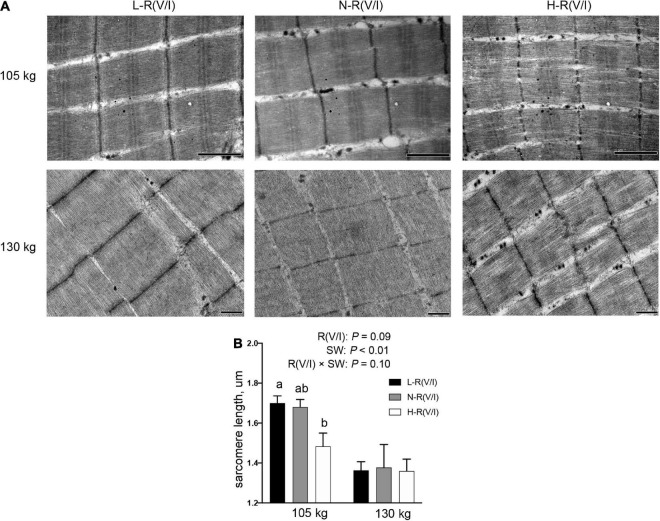
Effects of dietary R (V/I) and slaughter weight on the ultrastructure of the M. longissimus thoracis in finishing pigs. **(A)** The ultrastructure of longissimus dorsi from pigs treated with diet with three R (V/I) and slaughtered at 105 or 130 kg body weight. **(B)** Sarcomere length of the M. longissimus thoracis. Transmission electron microscopy images at original magnifications of × 30,000. Different letters represent significant differences among the groups (*P* < 0.05), *n* = 6.

The myofibril ultrastructure was shown in Figure 2. With slaughter weight increased, the M line located on the center of the A band looked vaguer, and the sarcomere length decreased (*P* < 0.01). The myofibrils of pigs fed with L-R(V/I) diet were tightly integrated with visible I and A band, and the Z lines, actin and myosin could be distinguished. However, the most decayed myofibrils structure could be found in the M. longissimus thoracis of pigs fed H-R(V/I) diet, in which myofibrils gradually fractured along the Z line. What’s more, along with dietary R (V/I) increased, the sarcomere length decreased in pigs slaughtered at 105 kg body weight, but no effect observed in pigs slaughtered at 130 kg body weight.

### Muscular Free Amino Acid and Total Amino Acid Profile

Muscular free amino acid profile was presented in Table 5. With slaughter weight increased, the amounts of muscular free lysine, tryptophan (*P* = 0.03), leucine, isoleucine, valine, alanine, arginine, and bitter amino acid were significantly increased (*P* < 0.01), while methionine, asparagic acid and glycine were decreased (*P* < 0.01). Besides, the amounts of muscular free phenylalanine, leucine, valine, alanine, asparagic acid and glutamic acid, glycine were significantly altered by dietary R (V/I) treatments (*P* < 0.05). Highest amounts of umami and sweet amino acid were observed in pigs fed N-R (V/I) diet compared with those in pigs fed H-R (V/I) and L-R (V/I) diet (*P* < 0.05). The significant interactions were observed between slaughter weight and dietary R (V/I) on muscular free methionine, phenylalanine, tryptophan, leucine, isoleucine, asparagic acid, proline, tyrosine and bitter amino acid (*P* < 0.05). The effects of dietary R (V/I) on these muscular free amino acids depended on the slaughter weight.

**TABLE 5:** Effect of dietary R (V/I) and slaughter weight on muscular free amino acid concentration in finishing pigs (nmol/g, *n* = 6).

Items	105 kg			130 kg			*P*-value		
	L-R (V/I)	N-R (V/I)	H-R (V/I)	L-R (V/I)	N-R (V/I)	H-R (V/I)	SW	R (V/I)	SW × R (V/I)
Histidine	77.78 ± 11.41	80.06 ± 5.46	73.99 ± 12.86	79.14 ± 4.02	74.30 ± 9.92	77.45 ± 6.13	0.92	0.79	0.49
Lysine	149.83 ± 43.75	176.84 ± 14.07	153.53 ± 23.25	209.84 ± 19.92	215.43 ± 33.51	198.70 ± 44.10	<0.01	0.36	0.75
Methionine	52.71 ± 10.74	66.20 ± 6.70	70.22 ± 2.58	57.72 ± 2.64	53.39 ± 3.92	47.69 ± 6.46	<0.01	0.25	<0.01
Phenylalanine	117.31 ± 10.49	135.00 ± 11.59	152.37 ± 8.91	135.71 ± 5.11	136.77 ± 15.23	122.27 ± 4.53	0.39	0.05	<0.01
Threonine	90.83 ± 22.18	101.98 ± 8.92	110.32 ± 13.02	102.51 ± 5.61	111.90 ± 16.11	104.87 ± 20.07	0.36	0.24	0.44
Tryptophan	42.28 ± 9.61	43.83 ± 6.34	48.18 ± 4.44	51.28 ± 0.92	51.52 ± 3.45	45.22 ± 2.84	0.03	0.91	0.05
Leucine	146.46 ± 20.81	176.35 ± 15.08	176.12 ± 19.01	213.78 ± 13.95	218.42 ± 22.30	170.26 ± 20.83	<0.01	0.03	<0.01
Isoleucine	60.74 ± 10.81	67.70 ± 6.66	92.37 ± 24.11	129.82 ± 8.12	126.68 ± 14.24	93.24 ± 11.01	<0.01	0.77	<0.01
Valine	174.58 ± 32.86	204.99 ± 8.34	215.15 ± 23.62	214.76 ± 6.22	245.07 ± 22.81	208.23 ± 30.18	<0.01	0.02	0.06
Alanine	1020.35 ± 116.66	1384.08 ± 176.83	1037.68 ± 107.94	1282.39 ± 93.03	1487.39 ± 37.02	1242.04 ± 228.08	<0.01	<0.01	0.46
Arginine	70.56 ± 11.41	76.71 ± 5.30	73.81 ± 7.09	92.20 ± 4.24	93.51 ± 15.47	82.65 ± 8.29	<0.01	0.31	0.36
Asparagic acid	36.04 ± 8.80	58.77 ± 18.92	24.80 ± 8.54	18.02 ± 3.10	17.33 ± 8.55	22.19 ± 11.12	<0.01	0.02	<0.01
Glutamic acid	141.70 ± 20.11	201.90 ± 66.94	161.97 ± 46.64	160.08 ± 33.68	185.93 ± 28.10	134.22 ± 15.25	0.57	0.03	0.41
Glycine	971.56 ± 191.61	1091.07 ± 189.13	814.81 ± 150.68	805.68 ± 39.83	737.75 ± 59.92	671.10 ± 35.96	<0.01	0.02	0.17
Proline	228.01 ± 28.04	259.85 ± 24.66	278.30 ± 24.17	272.92 ± 16.77	275.29 ± 22.69	236.83 ± 15.42	0.46	0.25	<0.01
Serine	198.61 ± 19.15	229.48 ± 24.96	213.88 ± 17.61	232.69 ± 21.25	223.51 ± 34.80	208.27 ± 12.29	0.39	0.34	0.11
Tyrosine	99.11 ± 17.95	111.08 ± 6.79	118.45 ± 1.91	112.87 ± 3.26	111.74 ± 8.76	104.30 ± 4395	0.98	0.34	0.01
Umami	177.74 ± 26.89	260.67 ± 84.87	186.76 ± 54.92	178.10 ± 36.54	203.27 ± 36.57	156.41 ± 17.48	0.10	0.02	0.42
Sweet	2833.77 ± 418.86	3448.40 ± 435.80	2823.66 ± 338.23	3120.79 ± 190.30	3296.35 ± 211.27	2870.04 ± 365.37	0.72	<0.01	0.37
Bitter	624.66 ± 89.00	713.09 ± 52.54	757.32 ± 68.53	821.23 ± 39.12	814.81 ± 81.20	697.87 ± 54.03	<0.01	0.28	<0.01

*L-R (V/I), low ratio of valine to isoleucine; N- R (V/I), normal ratio of valine to isoleucine; H-R (V/I), high ratio of valine to isoleucine; SW means slaughter weight; Umami = Aspartic acid + Glutamic acid; Sweet = Lysine + Threonine + Valine + Alanine + Glycine + Proline + Serine; Bitter = Arginine + Histidine + Isoleucine + Leucine + Methionine + Phenylalanine + Tyrosine.*

We determined amounts of protein-bound amino acids of fresh meat. Interestingly, we found that muscular contents of most protein-bound amino acids except tryptophan and arginine significantly decreased with the slaughter weight increased (*P* < 0.01) (Table 6). H-R (V/I) dietary treatment significantly decreased the content of lysine, methionine, threonine, alanine, asparagic acid, cysteine, glycine and serine compared with N-R (V/I) treatment (*P* < 0.05). There was no interaction between diet R (V/I) and slaughter weight.

**TABLE 6:** Effect of dietary R (V/I) and slaughter weight on total amino acid concentration of fresh meat in finishing pigs (% of freeze-drid meat, *n* = 6).

Items	105 kg			130 kg			*P*-value		
	L-R (V/I)	N-R (V/I)	H-R (V/I)	L-R (V/I)	N-R (V/I)	H-R (V/I)	SW	R (V/I)	SW × R (V/I)
Histidine	4.33 ± 0.07	4.32 ± 0.12	4.32 ± 0.19	3.93 ± 0.07	3.95 ± 0.09	3.89 ± 0.14	<0.01	0.86	0.86
Lysine	8.22 ± 0.06	8.18 ± 0.16	8.04 ± 0.09	7.44 ± 0.06	7.58 ± 0.13	7.44 ± 0.18	<0.01	0.05	0.16
Methionine	2.54 ± 0.03	2.54 ± 0.02	2.48 ± 0.03	2.32 ± 0.02	2.31 ± 0.04	2.29 ± 0.04	<0.01	<0.01	0.36
Phenylalanine	4.07 ± 0.16	4.00 ± 0.11	4.06 ± 0.17	3.38 ± 0.05	3.43 ± 0.04	3.37 ± 0.06	<0.01	0.98	0.43
Threonine	4.27 ± 0.06	4.26 ± 0.10	4.16 ± 0.04	3.91 ± 0.03	3.97 ± 0.07	3.90 ± 0.07	<0.01	0.03	0.32
Tryptophan	1.04 ± 0.02	1.04 ± 0.02	1.04 ± 0.01	1.05 ± 0.02	1.05 ± 0.02	1.04 ± 0.01	0.56	0.71	0.82
Leucine	7.47 ± 0.03	7.40 ± 0.15	7.36 ± 0.1	6.53 ± 0.08	6.60 ± 0.16	6.54 ± 0.19	<0.01	0.65	0.43
Isoleucine	4.51 ± 0.06	4.45 ± 0.07	4.46 ± 0.12	4.06 ± 0.05	4.06 ± 0.07	4.03 ± 0.04	<0.01	0.44	0.57
Valine	4.72 ± 0.11	4.68 ± 0.06	4.70 ± 0.14	4.20 ± 0.07	4.31 ± 0.07	4.22 ± 0.15	<0.01	0.75	0.29
Alanine	5.27 ± 0.08	5.28 ± 0.12	5.25 ± 0.11	5.09 ± 0.05	5.20 ± 0.08	5.03 ± 0.1	<0.01	0.07	0.22
Arginine	5.55 ± 0.04	5.57 ± 0.16	5.45 ± 0.12	5.48 ± 0.06	5.64 ± 0.11	5.54 ± 0.21	0.55	0.13	0.31
Asparagic acid	8.46 ± 0.04	8.41 ± 0.16	8.24 ± 0.1	7.76 ± 0.04	7.90 ± 0.16	7.72 ± 0.2	<0.01	0.02	0.24
Cysteine	0.99 ± 0.02	0.98 ± 0.02	0.96 ± 0.02	0.89 ± 0.02	0.89 ± 0.03	0.85 ± 0.02	<0.01	<0.01	0.75
Glutamic acid	12.73 ± 0.03	12.53 ± 0.33	12.44 ± 0.16	11.84 ± 0.13	12.01 ± 0.2	11.85 ± 0.29	<0.01	0.30	0.14
Glycine	3.93 ± 0.04	3.99 ± 0.14	3.92 ± 0.07	3.54 ± 0.04	3.65 ± 0.06	3.49 ± 0.07	<0.01	<0.01	0.42
Proline	3.87 ± 0.3	3.92 ± 0.17	3.82 ± 0.28	3.17 ± 0.19	3.51 ± 0.09	3.35 ± 0.06	<0.01	0.11	0.25
Serine	3.55 ± 0.04	3.55 ± 0.08	3.44 ± 0.05	3.30 ± 0.01	3.37 ± 0.05	3.22 ± 0.1	<0.01	<0.01	0.43
Tyrosine	2.88 ± 0.46	3.05 ± 0.18	2.87 ± 0.46	2.50 ± 0.07	2.53 ± 0.03	2.44 ± 0.07	<0.01	0.55	0.85

*L-R (V/I), low ratio of valine to isoleucine; N- R (V/I), normal ratio of valine to isoleucine; H-R (V/I), high ratio of valine to isoleucine; SW means slaughter weight.*

### Composition of Fatty Acid in the Longissimus Dorsi Muscle

Compared with pigs slaughtered at 105 kg, pigs slaughtered at 130 kg BW had higher total fatty acids, HH and composition of C14:0, C16:0, C16:1, C18:1n9c and MUFA, but lower IT, PUFA/SFA and composition of C14:1, C15:0, C17:0, C18:2n6c, C18:3n3, C21:0, C20:3n6, C20:4n6, C20:3n3, C20:5n3, C22:0, C24:0, C22:6n3, C24:1, PUFA, 6-PUFA, and 3-PUFA (*P* < 0.01) (Table 7).

**TABLE 7:** Effect of dietary R (V/I) and slaughter weight on fatty acids composition (% of total fatty acid) of fresh meat in finishing pigs (*n* = 6).

Items	105 kg			130 kg			*P*-value		
	L-R (V/I)	N-R (V/I)	H-R (V/I)	L-R (V/I)	N-R (V/I)	H-R (V/I)	SW	R (V/I)	SW × R (V/I)
Total fatty acids, % dried meat	47.06 ± 9.03	48.61 ± 16.42	52.88 ± 7.30	74.30 ± 8.23	68.27 ± 12.37	82.17 ± 9.66	<0.01	0.18	0.59
C10:0	0.15 ± 0.01	0.17 ± 0.01	0.14 ± 0.01	0.15 ± 0.01	0.15 ± 0.01	0.14 ± 0.01	0.21	<0.01	0.11
C12:0	0.13 ± 0.01	0.13 ± 0.01	0.13 ± 0.01	0.13 ± 0.01	0.14 ± 0.01	0.12 ± 0.01	0.65	0.29	0.09
C14:0	1.31 ± 0.10	1.32 ± 0.10	1.25 ± 0.10	1.44 ± 0.10	1.44 ± 0.10	1.36 ± 0.10	<0.01	0.21	0.98
C14:1	0.022 ± 0.003	0.034 ± 0.004	0.019 ± 0.003	0.021 ± 0.006	0.024 ± 0.007	0.013 ± 0.006	<0.01	<0.01	0.15
C15:0	0.04 ± 0.01	0.04 ± 0.01	0.037 ± 0.01	0.03 ± 0.01	0.026 ± 0.01	0.026 ± 0.01	<0.01	0.47	0.88
C16:0	25.22 ± 0.94	24.47 ± 0.94	25.08 ± 0.94	26.07 ± 0.94	26.15 ± 0.94	26.08 ± 0.94	<0.01	0.69	0.56
C16:1	2.97 ± 0.28	3.20 ± 0.28	3.06 ± 0.28	3.32 ± 0.28	3.44 ± 0.28	3.30 ± 0.28	0.02	0.40	0.90
C17:0	0.20 ± 0.04	0.18 ± 0.04	0.17 ± 0.04	0.14 ± 0.04	0.13 ± 0.04	0.12 ± 0.04	<0.01	0.047	0.82
C18:0	14.99 ± 0.64	14.28 ± 0.64	14.22 ± 0.64	14.4 ± 0.64	14.02 ± 0.64	14.07 ± 0.64	0.21	0.17	0.78
C18:1n9c	38.63 ± 2.05	38.18 ± 2.05	39.83 ± 2.05	41.91 ± 2.05	41.19 ± 2.05	43.84 ± 2.05	<0.01	0.07	0.85
C18:2n6c	11.69 ± 2.01	12.17 ± 2.01	11.39 ± 2.01	9.19 ± 2.01	9.72 ± 2.01	7.99 ± 2.01	<0.01	0.24	0.76
C18:3n3	0.33 ± 0.05	0.32 ± 0.05	0.32 ± 0.05	0.28 ± 0.05	0.27 ± 0.05	0.25 ± 0.05	<0.01	0.34	0.49
C20:0	0.20 ± 0.01	0.19 ± 0.01	0.21 ± 0.01	0.20 ± 0.01	0.20 ± 0.01	0.20 ± 0.01	0.89	0.24	0.13
C21:0	0.43 ± 0.07	0.51 ± 0.07	0.42 ± 0.07	0.32 ± 0.07	0.31 ± 0.07	0.31 ± 0.07	<0.01	0.12	0.12
C20:3n6	0.38 ± 0.05	0.48 ± 0.05	0.38 ± 0.05	0.25 ± 0.05	0.27 ± 0.05	0.23 ± 0.05	<0.01	0.02	0.19
C20:4n6	2.52 ± 0.35	3.31 ± 0.35	2.52 ± 0.35	1.71 ± 0.35	2.04 ± 0.35	1.54 ± 0.35	<0.01	<0.01	0.52
C20:3n3	0.068 ± 0.010	0.088 ± 0.018	0.072 ± 0.004	0.045 ± 0.002	0.042 ± 0.006	0.038 ± 0.003	<0.01	0.04	0.02
C20:5n3	0.065 ± 0.009	0.081 ± 0.015	0.08 ± 0.020	0.037 ± 0.004	0.042 ± 0.004	0.045 ± 0.008	<0.01	0.06	0.55
C22:0	0.08 ± 0.02	0.09 ± 0.02	0.077 ± 0.02	0.05 ± 0.02	0.054 ± 0.02	0.047 ± 0.02	<0.01	0.27	0.83
C24:0	0.42 ± 0.06	0.53 ± 0.06	0.43 ± 0.06	0.22 ± 0.06	0.25 ± 0.06	0.20 ± 0.06	<0.01	0.05	0.39
C22:6n3	0.076 ± 0.009	0.122 ± 0.028	0.083 ± 0.004	0.039 ± 0.004	0.049 ± 0.007	0.035 ± 0.005	<0.01	<0.01	0.01
C24:1	0.085 ± 0.013	0.099 ± 0.021	0.081 ± 0.006	0.035 ± 0.002	0.046 ± 0.007	0.035 ± 0.003	<0.01	0.01	0.74
SFA	43.17 ± 0.39	41.91 ± 0.39	42.16 ± 0.39	43.16 ± 0.39	42.88 ± 0.39	42.68 ± 0.39	0.26	0.26	0.65
MUFA	41.71 ± 2.31	41.51 ± 2.31	42.99 ± 2.31	45.29 ± 2.31	44.70 ± 2.31	47.18 ± 2.31	<0.01	0.11	0.87
PUFA	15.13 ± 2.46	16.57 ± 2.46	14.85 ± 2.46	11.55 ± 2.46	12.42 ± 2.46	10.13 ± 2.46	<0.01	0.13	0.84
6-PUFA	14.59 ± 2.39	15.96 ± 2.39	14.29 ± 2.39	11.15 ± 2.39	12.02 ± 2.39	9.76 ± 2.39	<0.01	0.13	0.84
3-PUFA	0.54 ± 0.08	0.61 ± 0.08	0.56 ± 0.08	0.40 ± 0.08	0.41 ± 0.08	0.37 ± 0.08	<0.01	0.14	0.34
n-6/n-3	27.17 ± 1.18	25.91 ± 1.18	25.68 ± 1.18	27.79 ± 1.18	29.6 ± 1.18	26.36 ± 1.18	0.08	0.26	0.30
PUFA/SFA	0.35 ± 0.06	0.40 ± 0.06	0.35 ± 0.06	0.27 ± 0.06	0.29 ± 0.06	0.24 ± 0.06	<0.01	0.12	0.77
IA	0.80 ± 0.02	0.76 ± 0.07	0.77 ± 0.02	0.81 ± 0.05	0.80 ± 0.03	0.80 ± 0.02	0.06	0.30	0.72
HH	1.39 ± 0.03	1.31 ± 0.12	1.34 ± 0.03	1.42 ± 0.08	1.41 ± 0.06	1.40 ± 0.04	0.01	0.25	0.59
IT	2.01 ± 0.09	2.10 ± 0.18	2.06 ± 0.08	1.93 ± 0.12	1.93 ± 0.11	1.96 ± 0.04	<0.01	0.63	0.62

*L-R (V/I), low ratio of valine to isoleucine; N- R (V/I), normal ratio of valine to isoleucine; H-R (V/I), high ratio of valine to isoleucine; SW means slaughter weight.*

Highest compositions of C14:1, C20:3n6, C20:4n6, C20:3n3, C24:0, C22:6n3 and C24:1 were observed in pigs fed N-R (V/I) diet compared with those in pigs fed H-R (V/I) and L-R (V/I) diet (*P* < 0.05). Meanwhile, L-R (V/I) and N-R (V/I) dietary treatments increased the composition of C10:0 and C17:0 compared with H-R (V/I) treatment. Interaction between R (V/I) and slaughter weight were observed for C20:3n3 and C22:6n3.

## Discussion

Though valine and isoleucine are essential amino acids for pigs, they have been found to exert opposite impact on meat quality in finishing pigs (3, 4). In addition, slaughter weight is generally supposed to be a key factor affecting meat quality. Therefore, in the present study, we explore influences of slaughter weight, dietary ratio of valine to isoleucine and their interaction on carcass traits, meat quality and fresh meat nutrition values.

As expected, we observed that backfat thickness, fat-free lean meat index of carcass and IMF content increased while crude protein content of meat decreased as slaughter weight increased from 105 to 130 kg, which was in line with previous studies for crossbred Duroc × Landrace × Yorkshire pigs (11, 22–25). It is worth noting that the effect of slaughter weight on carcass traits and meat quality varies among pig breeds. For example, intramuscular fat content was not affected by slaughter weight in Pietrain pigs (26), Swiss Yorkshire barrows (27), crossbred Landrace × Yorkshire (28) and crossbred (Landrace × Yorkshire) × (Pietrain × Yorkshire) pigs (29), but increased with increased slaughter weight in crossbred Yorkshire × (Landrace × Pietrain) pigs (30) and Hampshire × Yorkshire × Duroc pigs (31). In general, carcass backfat thickness increases as slaughter weight increased among pig breeds (11, 22, 23, 25, 27, 29–32). We also found that meat color indices (a*_45 *min*_ and b*_45 *min*_), drip loss and shear force of cooked meat increased as pig slaughter weight increased, while loin eye area of carcasses remained unchanged. The result of slaughter weight on meat color supported by previous studies, the M. longissimus thoracis of pigs slaughtered at heavy body weight was redder (higher a* value; *P* < 0.01) than that of pigs slaughtered at slight body weight in crossbred Duroc × Landrace × Yorkshire pigs (22, 23, 32), Yorkshire × (Landrace × Pietrain) pigs (30) and crossbred (Landrace × Yorkshire) × (Pietrain × Yorkshire) pigs (29). Interestingly, we notified that shear force value increased parallel to IMF content as pig slaughter weight increased. It suggested that shear force of cooked meat is not only correlated with IMF content especially in carcasses with significant gap of slaughter weights, but also correlated with other physiological characteristics of muscle, such as collagen structure, myofiber distribution and calpain enzyme activity as shown in previous studies (33, 34). It implied that optimal slaughter weight should be different for various meat quality indices.

According to previous studies (35), water distribution significantly affects water holding capacity (WHC) of fresh meat. The water distribution of fresh meat reflected by three fractions determined by NMR T2 relaxation analysis including bound water (*T*_21_), immobilized water (*T*_22_) and free water (*T*_23_). Besides, the free water reflects extra- myofibrillar water, which is mainly contributing to potential drip loss (36). Compared with immobilized water, the bound water only represented a minor part of the water present, and its change has limited impact on the WHC of meat. In the present study, the peak area ratio (%) of immobilized water (*T*_22_) was higher whereas the peak area ratio (%) of bound water (*T*_21_) was lower in pigs slaughtered at 130 kg BW than at 105 kg BW, as a consequence, the drip loss of fresh meat was higher in pigs slaughtered at 130 kg BW than pigs at 105 kg BW. And for the first time, we found that increasing slaughter weight reduces sarcomere length, which might also be partly responsible for the increased drip loss by the increased slaughter weight.

It is generally accepted that the fatty acid composition of intramuscular lipid in pork changed greatly with the increased slaughter weight (11). Consistently, we observed that the sum of MUFA increased but PUFA decreased as slaughter weight increased. Besides, we calculated health lipid indexes based on fatty acid composition of intramuscular lipid to evaluate the health quality of the muscle. The indices of atherogenicity (IA) and thrombogenicity (IT) have been introduced to characterize atherogenic and thrombogenic potential (20). The higher the ratio between hypocholesterolemic and hypercholesterolemic fatty acids (HH), the more the oil or fat is adequate to human nutrition (21). In our study, pigs slaughtered at 130 kg showed lower IT and higher HH, indicating that meat of pigs slaughtered at higher body weight may be beneficial to human health.

Compared with lighter pigs, heavier pigs exhibited higher IMF content and lower content of protein in the heavier pigs, which is in line with the previous study in crossbred Duroc × Landrace × Yorkshire pigs (23). However, in previous studies, it has been also observed that muscle protein content was not influenced with slaughter weight increased in crossbred Landrace × Yorkshire (28), crossbred (Landrace × Yorkshire) × (Pietrain × Yorkshire) pigs (29), and Swiss Yorkshire barrows (27). The inconsistency may be due to various pig breeds. Moreover, little attention was paid on the variation of amino acid profile caused by slaughter weight before. Here, we demonstrated for the first time that increased slaughter weight reduced most kinds of muscular total amino acid contents except for tryptophan and arginine, while increase muscular free amino acid contents, including lysine, tryptophan, leucine, isoleucine, valine, alanine, and arginine. The lower muscular total amino acids for the higher slaughter weight group could be attributed to its lower protein content and higher fat content in heavy pigs than those in light pigs.

The proportion of branched-chain amino acids could alter porcine fat mass (37). Particularly, backfat thickness was reduced in pigs fed diets supplemented with isoleucine compared with the control (38). Our previous studies have shown that dietary supplementation of isoleucine increase IMF content and water holding capacity of fresh meat in finishing pigs, while high level of dietary valine undermined water holding capacity (4). Consistently, in the present study, we observed that pigs fed H-R (V/I) diet had the weakest water holding capacity of fresh meat in all pigs. Water holding capacity are influenced by many factors, including pH, water distribution and muscle structure (4, 15, 39, 40). Here, we demonstrated that H-R (V/I) diet decreased ultimate pH value and sarcomere length but increased the proportion of free water (*T*_23_), consequently, undermined fresh meat WHC. Compared with pigs fed N-R (V/I) diet, pigs fed L-R(V/I) or H-R(V/I) diet had increased backfat thickness and IMF content, indicating that unbalanced dietary supply of valine and isoleucine (L-R(V/I) and H-R(V/I) diets) could lead to the obese in finishing pigs.

Although dietary R (V/I) exerted a secondary impact on muscular fatty acid profile compared with slaughter weight, we observed that balanced dietary valine and isoleucine supply (N-R (V/I) increased several PUFA, such as C20:3n6, C20:4n6, C20:3n3 and C22:6n3. In addition, we found that pigs received N-R (V/I) treatment increased muscular free amino acids that contribute to meat flavor, including the umami taste amino acids (aspartic acid and glutamic acid), sweet taste amino acids (glycine, alanine, valine), but did not affect bitter taste amino acids (methionine, isoleucine, tyrosine, phenylalanine, histidine, and arginine) compared with pigs received other two dietary treatments. Pigs fed the diet with N-R (V/I) had the highest nutritional value in terms of the muscular abundance of essential amino acids (lysine, methionine, and threonine) and non-essential amino acids (aspartic acid, cysteine, glycine, and serine).

We also observed significant interaction between dietary R(V/I) and slaughter weight on the last rib backfat thickness, fat-free lean index, and meat color. The effect of R(V/I) on these indicators weakened with the increase of slaughter weight, indicating that pigs with heavier slaughter weights are more resistant to the effected of dietary R(V/I) treatments.

## Conclusion

Increased slaughter weight markedly favored meat color and IMF content but undermined water holding capacity of fresh meat and tenderness of cooked meat in finishing crossbred Duroc × Landrace × Yorkshire pigs. Pigs received N-R (V/I) diet improved carcass traits and meat flavor-contributing amino acids at the cost of reduction in IMF content and increase in shear force of cooked meat compared with the pigs fed L-R (V/I) and H-R(V/I) diets. In particular, the effects of dietary R(V/I) on carcass traits and meat color were influenced by slaughter weight.

## Data Availability Statement

The original contributions presented in this study are included in the article/supplementary material, further inquiries can be directed to the corresponding author.

## Ethics Statement

The animal study was reviewed and approved by the Institutional Animal Care and Use Committee of China Agricultural University.

## Author Contributions

DX performed the animal experiments, analyzed the data, and wrote the manuscript. JY, XZ, and DX contributed to the experimental design and manuscript modification. YW was mainly responsible for the sample collection and data curation. EY, LH, LW, CM, and PZ carried out meat quality test. All authors approved the manuscript.

## Conflict of Interest

The authors declare that the research was conducted in the absence of any commercial or financial relationships that could be construed as a potential conflict of interest.

## Publisher’s Note

All claims expressed in this article are solely those of the authors and do not necessarily represent those of their affiliated organizations, or those of the publisher, the editors and the reviewers. Any product that may be evaluated in this article, or claim that may be made by its manufacturer, is not guaranteed or endorsed by the publisher.

## Abbreviations

R(V/I): ratio of valine to isoleucine
BCAA: branched-chain amino acids
MUFA: monounsaturated fatty acids
BW: body weight
SID: standard ileal digestible
IMF: intramuscular fat
IA: atherogenic index
IT: thrombogenic index
HH: ratio between hypocholesterolemic and hypercholesterolemic fatty acids
PUFA: polyunsaturated fatty acids.
